# Clustered regularly interspaced short palindromic repeats/CRISPR-associated protein and hairy roots: a perfect match for gene functional analysis and crop improvement

**DOI:** 10.1016/j.copbio.2022.102876

**Published:** 2023-02

**Authors:** Josefa M Alamillo, Cristina M López, Félix J Martínez Rivas, Fernando Torralbo, Mustafa Bulut, Saleh Alseekh

**Affiliations:** 1 Departamento de Botánica, Ecología y Fisiología Vegetal, Grupo de Fisiología Molecular y Biotecnología de Plantas, Campus de Excelencia Internacional Agroalimentario, CEIA3, Campus de Rabanales, Edif. Severo Ochoa, Universidad de Córdoba, 14071 Córdoba, Spain; 2 Max-Planck-Institute of Molecular Plant Physiology, 14476 Potsdam-Golm, Germany; 3 Institute of Plants Systems Biology and Biotechnology, Plovdiv, Bulgaria

## Abstract

Clustered regularly interspaced short palindromic repeats/CRISPR-associated protein (CRISPR/Cas) gene editing has become a powerful tool in genome manipulation for crop improvement. Advances in omics technologies, including genomics, transcriptomics, and metabolomics, allow the identification of causal genes that can be used to improve crops. However, the functional validation of these genetic components remains a challenge due to the lack of efficient protocols for crop engineering. Hairy roots gene editing using CRISPR/Cas, coupled with omics analyses, provide a platform for rapid, precise, and cost-effective functional analysis of genes. Here, we describe common requirements for efficient crop genome editing, focused on the transformation of recalcitrant legumes, and highlight the great opportunities that gene editing in hairy roots offers for future crop improvement.

## Highlights

• CRISPR/Cas9 gene editing is a powerful tool in functional analysis for crop improvement.
• The lack of efficient transformation protocols hinders CRISPR/Cas9 use in pulse crops.
• Omics- analyses of CRISPR/Cas edited hairy roots speeds up positive traits discovery.

## Graphical Abstract

**Figure:**
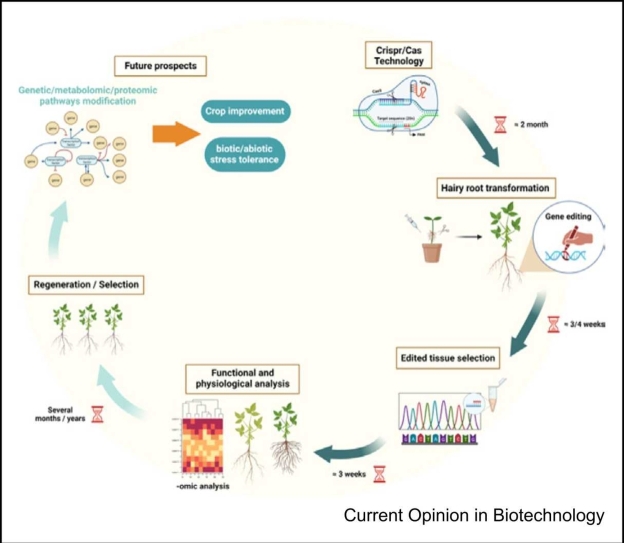

**Current Opinion in Biotechnology** 2023, **79**:102876

This review comes from a themed issue on **Plant Biotechnology**

Edited by **Alisdair Fernie** and **Jianbing Yan**

For complete overview of the section, please refer to the article collection, “Plant Biotechnology (2023)”

https://doi.org/10.1016/j.copbio.2022.102876

0958-1669/© 2022 The Author(s). Published by Elsevier Ltd. This is an open access article under the CC BY license (http://creativecommons.org/licenses/by/4.0/).

## Introduction

Global population growth and worsening climatic conditions are leading to an increased demand for more resilient crops [1]. Traditional breeding programs achieved great success in the development of high-yielding elite crop varieties. Nonetheless, it has been used to a less extent to improve abiotic stress resistance to face the current scenario of climate change. In crop plants, valuable alleles retained in local landraces or wild relatives have been introduced into elite cultivars by laborious and time-consuming traditional crop breeding 2, 3. These valuable traits maintained in local landraces often differ from elite cultivars in single-nucleotide polymorphisms, insertions, or deletions of gene fragments. The development of Clustered regularly interspaced short palindromic repeats/CRISPR-associated protein (CRISPR/Cas) genome editing technology, which allows precise genetic manipulation, has become an increasingly powerful tool for plant research and crop improvement 4, 5•. Targeted manipulation of plant genomes facilitated the incorporation of beneficial traits and/or the elimination of undesirable ones in a more precise and faster way compared with traditional breeding. These manipulations have already reported notable advances, such as improved nitrogen use efficiency in rice and wheat 6, 7, low-gluten wheat [8], and low-phytate soybean [9]. Although gene editing has been implemented in some legumes, such as soybean, lotus, and Medicago (Table 1), the entire potential of CRISPR/Cas9 technology is far from being exploited in many legume crops, due to the lack of efficient transformation-regeneration protocols. Nevertheless, *Rhizobium rhizogenes-*mediated CRISPR/Cas9 hairy root transgenic system provides a powerful biotechnological tool to study the functional genomics for pulse crop improvement.

**Table 1:** Gene-edited legumes by CRISPR/Cas.

Plant species	Delivery method	Targeted genes	Transformation^a^	Results	Reference
*Glycine max*	*R. rhizogenes*	*GmFEI, GmSHR and bar*	HR	Successful edition of both endogenous and exogenous genes	[25]
*Glycine max*	Biolistic transformation	*Glyma.08G116300.1*	TP	T1-inherited mutations in 30K gene	[47]
*Glycine max*	*A. tumefaciens*	*GmPIN*	TP	Demonstrate the importance of GmPIN1 in leaf petiole angle using stable gene-edited soybean plants	[48]
*Glycine max*	*A. tumefaciens*	*GmNN1/FT2a*	TP	Altered soybean nodulation, plant growth, and N nutrition	[49]
*Glycine max*	*R. rhizogenes*	*FAD2-A and Glyma10g42470*	HR	CRISPR/LbCpf1-induced large chromosome segment deletions	[50]
*Glycine max*	*R. rhizogenes*	*GmIPK1 and GmIPK2*	HR	Successful gene editing	[51]
*Glycine max*	*A. tumefaciens*	*GmIPK1*	TP	Developed soybean gene-edited plants with low PA content	[9]
*Arachis hypogaea L.*	*R. rhizogenes*	*AhNFR1 and AhNFR5*	HR	Validated the function of AhNFR5 genes in nodule formation	[46]
*Arachis hypogaea L.*	*R. rhizogenes*	*FAD2*	HR	Successful gene editing	[52]
*Arachis hypogaea L.*	*R. rhizogenes*	*FAD2*	HR	Successful gene editing	[53]
*Lotus japonicus*	*A. tumefaciens* and *R. rhizogenes*	*LjSYMRK and LjLb*	HR and TP	Targeted single and multiple SNF genes by hairy root transformation or in stable TP	[54]
*Lotus japonicus*	*R. rhizogenes*	*LjLb*	TP and HR	Biochemical, molecular, and physiological characterization of stable mutants	[55]
*Vigna unguiculata*	*A. tumefaciens*	*Vu-SPO11*	TP	Successful gene editing	[34]
*Vigna unguiculata*	*R. rhizogenes*	*VuSYMRK*	HR	Successful gene editing	[56]
*Vigna unguiculata*	*A. tumefaciens*	*VuSPO11–1*	TP	Successful gene editing	[57]
*Cicer arietinum*	*PEG4000*	*4CL and RVE7*	Protoplast	Successful gene editing	[58]
*Medicago truncatula*	*R. rhizogenes*	*MtPDS and MtCOMT*	HR	Obtained biallelic or homozygous mutated lines via fast-growing hairy root system	[33]
*Medicago sativa*	*A. tumefaciens*	*PHO2*	TP	Generation of mutants that hyperaccumulated Pi	[59]
*Phaseolus vulgaris*	*R. rhizogenes*	*XMPP, GSDA, NSH1, NSH2, and XDH*	HR	Identify essential metabolites for ureide biosynthesis	[42]

a HR: hairy roots; TP: transgenic plants.

In this review, we present an updated view of the development of CRISPR/Cas9 gene-editing tools to use in plant engineering, we describe the requirements and challenges that this technology still poses for agronomic use, and highlight the ample opportunities that available tools can offer to plant scientists for future crop improvements with a focus on legume species.

## Development of clustered regularly interspaced short palindromic repeats/CRISPR-associated protein genome editing tools

Genome editing technology began more than three decades ago with the induction of targeted deoxyribonucleic acid (DNA) double-strand breaks (DSB) by programmable meganucleases, zinc finger nucleases (ZFNs), and transcription activator-like effector nucleases (TALENs). But it is the most recent tool, based on ribonucleic acid (RNA)-guided nuclease CRISPR/Cas system, which has broadened the use of gene-editing technology to unprecedented horizons.

**Figure 1:**
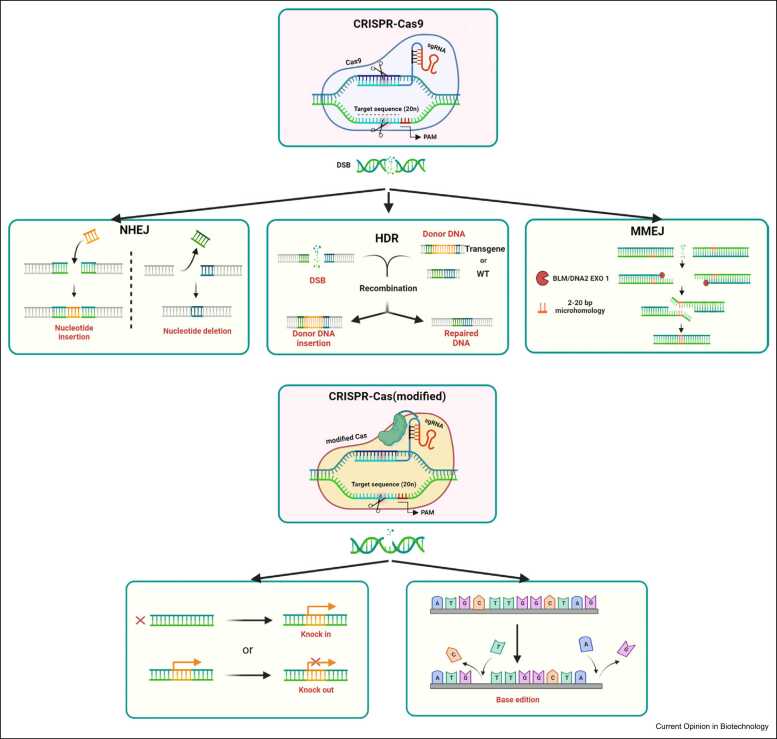
CRISPR/Cas gene editing and DNA repair mechanisms. Canonical Cas proteins use an RNA guide with 20 nt complementary to target DNA sequences adjacent to the 3-nt PAM (NGG in Cas9) sequence. Cas nucleases cause DSB in the DNA, which could be repaired by NHEJ or by HDR. NHEJ repair produces insertions or deletions, while HDR uses recombination with a DNA template whose ends are homologous to the break ends. This error-free DNA repair copies the DNA template from the wild-type (WT) or, from any supplied sequence, as transgene if the new sequence carries homologies at its ends. Cas variants, lacking activity of one or the two nuclease domains, can be used to directly write the genome. Created with BioRender.com.

The CRISPR/Cas evolved as an adaptive defense from invading viruses and plasmids in *archaea* and bacteria. The CRISPR repeats are transcribed into RNAs and processed into smaller pieces (crRNAs) that bind to a transacting RNA (tracrRNA) scaffold forming a complex that recruits Cas nuclease protein. Cas targets a specific short sequence, namely protospacer-adjacent motif (PAM), and a complementary sequence to the 5′-end of the crRNA. Cas9 from *Streptococcus pyogenes,* the most used Cas protein, recognizes a 20-nt-long complementary crRNA sequence, adjacent to NGG as PAM site, and cuts the two strands of the target DNA 10•, 11. The easier design of RNA guides compared with the need of engineering DNA-binding motives in ZFNs and TALENs has led to the wide adoption of CRISPR/Cas9 for targeted DSBs on genome-engineering applications in animals and plants [12]. Moreover, new Cas proteins have been identified with different target requirements in various prokaryotes, and multiple Cas variants have been developed, including nickase nCas, deactivated dCas, and Cas proteins fused to various effector domains for transcriptional activation (CRISPRa), interference (CRISPRi), or for precise sequence editing (base editing and prime editing) that write new genetic information into a specified DNA site (Figure 1) 13, 14, 15, 16••, 17, 18, 19, raising almost unlimited gene-editing applications.

Nonhomologous end joining (NHEJ) and homology-directed repair (HDR) are the most prevalent DSB repair mechanisms (Figure 1). NHEJ often results in one to various nucleotide insertions or deletions (indels). DSB can be also repaired by microhomology-mediated end joining (MMEJ) and single-strand annealing. These alternative systems are also error-prone because the accurately repaired sequence will be again recognized by the nuclease and recleaved until it acquires an indel, which can no longer be targeted. Instead of error-prone NHEJ, accurate repair by HDR occurs if a homologous DNA is available to serve as a repair template. However, although it holds the greatest potential for the insertion of desired sequences, HRD is a very low-efficiency process in most cells 12, 20.

## Limitations and requirements for efficient crop genome editing

Plant genetic transformation and gene-editing technologies have facilitated plant biology studies and crop improvement. However, transformation and regeneration of stably transformed plants remain a challenge in most of the crops, including legumes. The recent development of CRISPR/Cas gene editing (Figure 1) significantly increased our ability to manipulate plant genomes for future crop improvements.

CRISPR/Cas9 gene editing relies on the availability of high-quality genome sequences, which requires a careful selection of guide RNAs (gRNAs) to effectively edit the target genes. Multiple bioinformatic tools have been developed to scan genomic sequences, choose the best target sites, and predict nondesirable off-target mutations. However, large genome sizes, polyploidies, and genetic diversity in plants set out considerable difficulties for whole-genome sequencing of many important crops, and often the desired genotype is not sequenced. Nonetheless, already-known genomic sequences from related genotypes could be used for some applications as gene knockdown that frequently targets exonic regions, which present high sequence conservation within genotypes. However, the use of sequence homology from close genotypes is not advised to target regulatory and noncoding sequences.

**Figure 2:**
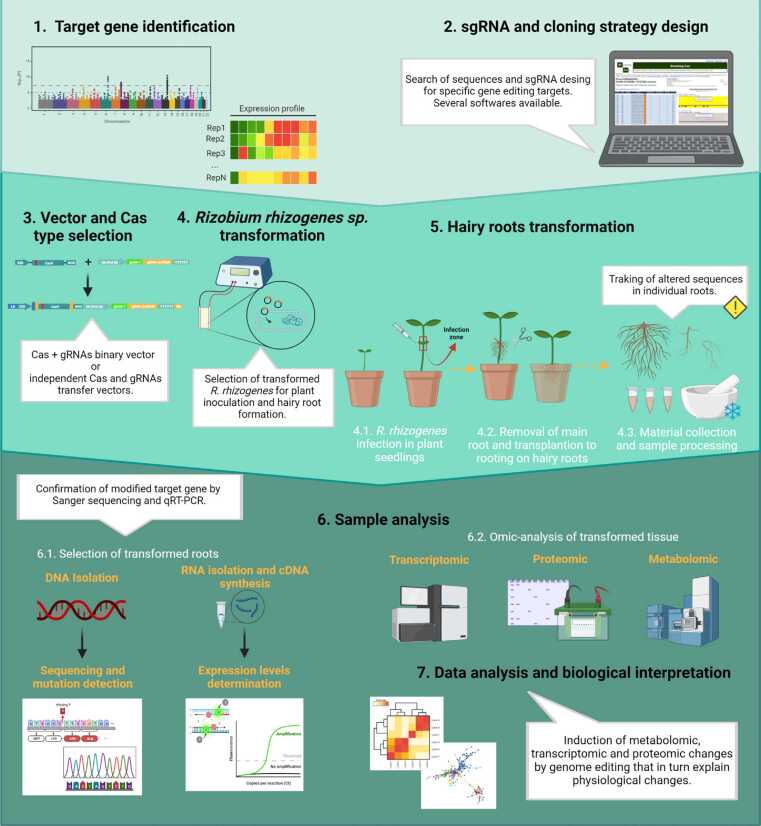
Hairy root gene-editing workflow. Gene editing using CRISPR/Cas in plants involves different steps. Among them, the following stand out: (1) the identification of the gene to be edited, (2) the design of the gRNAs and primers for the cloning, (3) the choice of the appropriate Cas protein, (4) transformation of *R. rhizogenes* strain, (5) hairy root transformation, (6) sample analysis and mutation identification, and (7) interpretation of the omics and physiological changes generated in the successfully edited tissue. (cDNA, complementary deoxyribonucleic acid; Cas, cutting enzyme). Created with BioRender.com.

Once a single-guide RNA (sgRNA) is designed, it should be flanked by appropriate promotor and terminator sequences and fused to the tracrRNA scaffold for Cas protein binding. In most CRISPR/Cas9 systems, gRNAs are driven by RNA polymerase III U6 or U3 promoters and terminator sequences. Strong cauliflower mosaic virus 35S or plant ubiquitin promoters have been often used to direct Cas9 expression, although promoters that would also be active at early developmental stages, as the ribosomal protein S5 (RPS5A), have been used to improve Cas9 editing 21, 22. Interestingly, RPS5 promoter is also active in the division zone of the root meristem, suggesting that it can be used to induce mutations in hairy roots. Typical transfer constructs comprise the Cas9, gRNA expression cassettes, and antibiotic or herbicide resistance genes, ideally within the same plasmid (Figure 2). In addition, plasmids are available with all the DNA transfer elements and a multiple polylinker to insert one or several gRNA expression units. The short length of gRNAs and strict PAM requirements often limit the editing of specific targets, which can be overcome using any of the available Cas protein variants 13, 15, 16••.

The most frequently used methods to deliver constructs to plant cells are (i) infection with *Agrobacterium tumefaciens,* (ii) direct transfer of DNA-coated particles using gene gun, and (iii) polyethylene glycol-treated protoplast. *Agrobacterium*-mediated gene transfer is preferred in dicots due to its higher precision and single-construct insertion in the genome. However, biolistic or protoplast transformation could be used for plants that are resistant to *A. tumefaciens*. An alternative is the transformation of hairy roots using *Rhizobium rhizogenes* (formerly *Agrobacterium rhizogenes*). This approach has been developed for the transformation of plants that are resistant to *A. tumefaciens,* as some potato cultivars [23] and grain legumes 24•, 25. Moreover, biolistic delivery into plant cells of *in vitro* preassembled ribonucleoprotein complexes of purified Cas protein and *in vitro*-transcribed gRNAs is emerging as a DNA-free gene-editing tool that avoids the integration of foreign DNA fragments 26, 27.

Next to be considered is the low incorporation of the desired alleles by genome editing in plants. DSB activates the cell’s DNA repair machinery that involves ligation of the breaks either by NHEJ and MMEJ, which may introduce indels; or by HDR, which creates precise sequence repair by using the identical unbroken DNA sequence of the pair chromatid or new DNA with homologous ends as templates. While CRISPR/Cas9-targeted mutagenesis have been used in many plants, including legumes (Table 1), HR-mediated targeted insertion remains a big challenge. This is probably because it operates only at the S and G2 phases of the cell cycle 20, 28, 29, but also due to the difficulty of delivering template DNAs to plant cells surrounded by semirigid cell walls. Nonetheless, successful examples of HR (gene targeting) in different crop species have been achieved 6, 30. To increase HR efficiency, several strategies have been developed, such as using cell-type-specific Cas nucleases, use of viral self-amplified vectors to increase the amount of template, or inhibiting NHEJ and/or stimulating HR 22, 28, 31. Although there are still difficulties, we believe that they could be soon overcome by numerous laboratories involved in gene targeting in plants.

The most important limitation of crop improvement using gene-editing tools is the lack of suitable protocols for plant regeneration from somatic transformed cells for many crops [32]. Antibiotic or herbicide resistance and/or marker green fluorescen protein (GFP) or β-glucuronidase (GUS) genes are included to ease the selection of transformed cells. However, the growth of transformed cells in selection media does not always imply a successful editing of the target gene. CRISPR–Cas9 gene editing has variable efficiency depending on the gRNAs used and the actual activation of the cell DNA repair mechanisms. Therefore, cells incorporating the transfer construct would survive selection, regardless of whether the target sequences are edited or not, often regenerating chimeric plants from both mutated and nonmutated cells, that hide the editing effects 28, 33. Moreover, biallelic or homozygous mutants by CRISPR–Cas9 could be only recovered after growing and screening a large number of plants, which undoubtedly requires more time and resources.

Screening for highly efficient editing sgRNA/Cas constructs, before performing genetic transformation, is usually done using plant protoplast. However, *in vitro* regeneration from protoplast is still an important bottleneck in crops that are considered recalcitrant to regeneration and/or transformation. Last, identifying and tracking modifications induced by genome editing is necessary. There are many methods for detecting genomic modifications, and the choice could depend on the information required. Tracking of mutations can be done by PCR analysis of decay of aberrant transcript expression, PCR amplification followed by restriction enzyme digestion and electrophoresis for large indels, and Sanger or massive sequencing among others.

## Current applications of hairy root transformation by clustered regularly interspaced short palindromic repeats/CRISPR-associated protein gene editing

Hairy root transformation with *R. rhizogenes* is more efficient, rapid, and simple than with *A. tumefaciens* infection. Upon transformation, it gives rise to hairy root syndrome in which chimeric composite plants with transgenic roots and nontransgenic shoots are generated in just a few weeks (Figure 2). Although the main limitation of hairy root transformation is its nonheritable chimeric nature, this could still be a valuable method for rapid functional analysis of genes that have important functions in roots, such as legume nodulation, mineral nutrition, or interactions with their environment [34]. Valuable information for the whole plant can be also obtained by phenotyping the chimeric hairy roots on a systemic level and thus, is often preferred to other transformation systems [35]. Furthermore, it allows for functional validation in nonregenerable species or those that need a long time to regenerate, such as pigeon pea, apple, aspen, and eucalyptus, among others 36•, 37, 38. On the other hand, hairy roots can be used to screen the effects of multiple mutations, since only a small number of endodermal founder cells at the periphery of the parental root are inherited and transfer their genetic materials to the newly formed lateral branches [39]. These newly obtained lateral roots should be considered as libraries bearing different edits produced by the CRISPR/Cas9 system. Therefore, screening for the mutated hairy roots must be done before further analysis (Figure 2). Plants regenerated from selected edited hairy roots could result in the fast selection of biallelic or homozygous CRISPR/Cas9-mutated lines without the need to increase the numbers of infected explants or prolonged sexual propagation [33].

## Application of metabolomic analysis of clustered regularly interspaced short palindromic repeats/CRISPR-associated protein-edited hairy roots

Plants produce a wide range of structurally and functionally diverse metabolites with different roles in plant growth and development and in plant response to continually changing environmental conditions and abiotic and biotic stresses [40]. In addition, they provide a rich source of numerous bioactive compounds that have been extensively employed in traditional medicine and crop nutritional quality. Even though hundreds of thousands of plant metabolites have been identified across the plant kingdom, the specific function of most plant metabolites has not yet been characterized. *R. rhizogenes*-mediated hairy root CRISPR/Cas9 transformation provides an efficient alternative tool for rapid and large-scale gene functional analysis in plants [40]. The gene-edited hairy root approach is ideal for dissecting the function of multicopy gene families common in plants, by knocking out of specific genes with CRISPR/Cas9 technology and analyzing the edited roots through metabolomic or transcriptomic approaches. That said, the method has been successfully used to functional analysis of different genes involved in plant metabolism [41]. For instance, the potential of coupling CRISPR/Cas9 and metabolomics has been able to characterize and update complex routes such as the ureide biosynthesis in common bean [42], which is of great importance for future breeding programs in this species. Among other examples, in tomato and potato, CRISPR/Cas9-mediated mutagenesis has been successfully used to validate genes involved in steroidal glycoalkaloid (SGA) accumulation 35•, 43. These studies showed the powerful hairy roots CRISPR/Cas9 and metabolomics combination to address gene functions to improve crop species.

While recent advances in plant-culture regeneration, gene-editing, and machine learning technologies [44] are expected to tackle the bottlenecks associated with transformation and regeneration procedures for the recalcitrant crops. In the meantime, transitory or chimeric transformation meditated by *R. rhizogenes* combined with CRISPR/Cas9 system has been proven as an excellent technique to generate gene-edited hairy roots 41•, 45. Although the main limitation of transgenic hairy roots is the lack of inheritability of the mutation into the progeny, it provides meaningful resources to decipher the functions of target genes before investing large amounts of time and resources in the generation of transgenic plants (TP) 25, 46.

## Author contributions

JMA and SA: Conceptualization, first draft preparation. CMLV, FJMR, and FT designed and created the figures. All authors contributed to MS writing – review & editing. All authors have read and approved the final version of the paper.

## Conflict of interest statement

The authors declare that they have no known competing financial interests or personal relationships that could have appeared to influence the work reported in this paper.

## Data Availability

Data will be made available on request.

## References

[1] Pereira A. (2016). Plant abiotic stress challenges from the changing environment. Front Plant Sci.

[2] Renzi J.P., Coyne C.J., Berger J., von Wettberg E., Nelson M., Ureta S., Hernández F., Smýkal P., Brus J. (2022). How could the use of crop wild relatives in breeding increase the adaptation of crops to marginal environments?. Front Plant Sci.

[3] Coyne C.J., Kumar S., von Wettberg E.J.B., Marques E., Berger J.D., Redden R.J., Ellis T.H.N., Brus J., Zablatzká L., Smýkal P. (2020). Potential and limits of exploitation of crop wild relatives for pea, lentil, and chickpea improvement. Legum Sci.

[4] Fernie A.R., Yan J. (2019). De novo domestication: an alternative route toward new crops for the future. Mol Plant.

[5•] Zsögön A., Čermák T., Naves E.R., Notini M.M., Edel K.H., Weinl S., Freschi L., Voytas D.F., Kudla J., Peres L.E.P. (2018). De novo domestication of wild tomato using genome editing. Nat Biotechnol.

[6] Li J., Zhang X., Sun Y., Zhang J., Du W., Guo X., Li S., Zhao Y., Xia L. (2018). **Efficient allelic replacement in rice by gene editing: a case study of the*****NRT1.1B*****gene**. J Integr Plant Biol.

[7] Zhang J., Zhang H., Li S., Li J., Yan L., Xia L. (2021). Increasing yield potential through manipulating of an ARE1 ortholog related to nitrogen use efficiency in wheat by CRISPR/Cas9. J Integr Plant Biol.

[8] Sánchez-León S., Gil-Humanes J., Ozuna C.V., Giménez M.J., Sousa C., Voytas D.F., Barro F. (2018). Low-gluten, nontransgenic wheat engineered with CRISPR/Cas9. Plant Biotechnol J.

[9] Song J.H., Shin G., Kim H.J., Lee S.B., Moon J.Y., Jeong J.C., Choi H., Kim I.A., Song H.J., Kim C.Y. (2022). **Mutation of*****GmIPK1*****gene using CRISPR/Cas9 reduced phytic acid content in soybean seeds**. Int J Mol Sci.

[10•] Jinek M., Chylinski K., Fonfara I., Hauer M., Doudna J.A., Charpentier E. (2012). A programmable dual-RNA-guided DNA endonuclease in adaptive bacterial immunity. Science.

[11] Doudna J.A., Charpentier E. (2014). The new frontier of genome engineering with CRISPR-Cas9. Science.

[12] Chandrasegaran S., Carroll D. (2016). Origins of programmable nucleases for genome engineering. J Mol Biol.

[13] Cebrian-Serrano A., Davies B. (2017). CRISPR-Cas orthologues and variants: optimizing the repertoire, specificity and delivery of genome engineering tools. Mamm Genome.

[14] Gaudelli N.M., Komor A.C., Rees H.A., Packer M.S., Badran A.H., Bryson D.I., Liu D.R. (2017). Programmable base editing of T to G C in genomic DNA without DNA cleavage. Nature.

[15] Ma D., Xu Z., Zhang Z., Chen X., Zeng X., Zhang Y., Deng T., Ren M., Sun Z., Jiang R. (2019). Engineer chimeric Cas9 to expand PAM recognition based on evolutionary information. Nat Commun.

[16••] Anzalone A.V., Randolph P.B., Davis J.R., Sousa A.A., Koblan L.W., Levy J.M., Chen P.J., Wilson C., Newby G.A., Raguram A. (2019). Search-and-replace genome editing without double-strand breaks or donor DNA. Nature.

[17] Bhowmik P., Konkin D., Polowick P., Hodgins C.L., Subedi M., Xiang D., Yu B., Patterson N., Rajagopalan N., Babic V. (2021). CRISPR/Cas9 gene editing in legume crops: opportunities and challenges. Legum Sci.

[18] Xin C., Yin J., Yuan S., Ou L., Liu M., Zhang W., Hu J. (2022). Comprehensive assessment of miniature CRISPR-Cas12f nucleases for gene disruption. Nat Commun.

[19] Zhang R.X., Li B.B., Yang Z.G., Huang J.Q., Sun W.H., Bhanbhro N., Liu W.T., Chen K.M. (2022). Dissecting plant gene functions using CRISPR toolsets for crop improvement. J Agric Food Chem.

[20] Rozov S.M., Permyakova N.V., Deineko E.V. (2019). The problem of the low rates of CRISPR/Cas9-mediated knock-ins in plants: approaches and solutions. Int J Mol Sci.

[21] Tsutsui H., Higashiyama T. (2017). **PKAMA-ITACHI vectors for highly efficient CRISPR/Cas9-mediated gene knockout in*****Arabidopsis thaliana***. Plant Cell Physiol.

[22] Wolter F., Klemm J., Puchta H. (2018). **Efficient in planta gene targeting in Arabidopsis using egg cell-specific expression of the Cas9 nuclease of*****Staphylococcus aureus***. Plant J.

[23•] Butler N.M., Jansky S.H., Jiang J. (2020). First-generation genome editing in potato using hairy root transformation. Plant Biotechnol J.

[24•] Estrada-Navarrete G., Alvarado-Affantranger X., Olivares J.E., Guillén G., Díaz-Camino C., Campos F., Quinto C., Gresshoff P.M., Sanchez F. (2007). **Fast, efficient and reproducible genetic transformation of*****Phaseolus*****spp. by*****Agrobacterium rhizogenes***. Nat Protoc.

[25] Cai Y., Chen L., Liu X., Sun S., Wu C., Jiang B., Han T., Hou W. (2015). CRISPR/Cas9-mediated genome editing in soybean hairy roots. PLoS One.

[26] Liang Z., Chen K., Li T., Zhang Y., Wang Y., Zhao Q., Liu J., Zhang H., Liu C., Ran Y. (2017). Efficient DNA-free genome editing of bread wheat using CRISPR/Cas9 ribonucleoprotein complexes. Nat Commun.

[27] Kumagai Y., Liu Y., Hamada H., Luo W., Zhu J., Kuroki M., Nagira Y., Taoka N., Katoh E., Imai R. (2022). Introduction of a second “Green Revolution” mutation into wheat via in planta CRISPR/Cas9 delivery. Plant Physiol.

[28] Huang T.K., Puchta H. (2019). CRISPR/Cas-mediated gene targeting in plants: finally a turn for the better for homologous recombination. Plant Cell Rep.

[29] Permyakova N.V., Marenkova T.V., Belavin P.A., Zagorskaya A.A., Sidorchuk Y.V., Deineko E.V. (2022). CRISPR/Cas9-Mediated targeted DNA integration: rearrangements at the junction of plant and plasmid DNA. Int J Mol Sci.

[30] Gil-Humanes J., Wang Y., Liang Z., Shan Q., Ozuna C.V., Sánchez-León S., Baltes N.J., Starker C., Barro F., Gao C. (2017). High-efficiency gene targeting in hexaploid wheat using DNA replicons and CRISPR/Cas9. Plant J.

[31] Wang M., Lu Y., Botella J.R., Mao Y., Hua K., kang Z.J. (2017). Gene targeting by homology-directed repair in rice using a geminivirus-based CRISPR/Cas9 system. Mol Plant.

[32] Hnatuszko-Konka K., Kowalczyk T., Gerszberg A., Wiktorek-Smagur A., Kononowicz A.K. (2014). ***Phaseolus vulgaris*** — **recalcitrant potential**. Biotechnol Adv.

[33] Zhang H., Cao Y., Zhang H., Xu Y., Zhou C., Liu W., Zhu R., Shang C., Li J., Shen Z. (2020). **Efficient generation of CRISPR/Cas9-mediated homozygous/biallelic*****Medicago truncatula*****mutants using a hairy root system**. Front Plant Sci.

[34•] Che P., Chang S., Simon M.K., Zhang Z., Shaharyar A., Ourada J., O’Neill D., Torres-Mendoza M., Guo Y., Marasigan K.M. (2021). Developing a rapid and highly efficient cowpea regeneration, transformation and genome editing system using embryonic axis explants. Plant J.

[35•] Swinnen G., De Meyer M., Pollier J., Molina-Hidalgo F.J., Ceulemans E., Venegas-Molina J., De Milde L., Fernández-Calvo P., Ron M., Pauwels L. (2022). The basic helix-loop-helix transcription factors MYC1 and MYC2 have a dual role in the regulation of constitutive and stress-inducible specialized metabolism in tomato. New Phytol.

[36•] Meng D., Yang Q., Dong B., Song Z., Niu L., Wang L., Cao H., Li H., Fu Y. (2019). Development of an efficient root transgenic system for pigeon pea and its application to other important economically plants. Plant Biotechnol J.

[37] Triozzi P.M., Schmidt H.W., Dervinis C., Kirst M., Conde D. (2021). **Simple, efficient and open-source CRISPR/Cas9 strategy for multi-site genome editing in*****Populus tremula*****×*****alba***. Tree Physiol.

[38] Ployet R., Veneziano Labate M.T., Regiani Cataldi T., Christina M., Morel M., San Clemente H., Denis M., Favreau B., Tomazello Filho M., Laclau J.P. (2019). **A systems biology view of wood formation in*****Eucalyptus grandis*****trees submitted to different potassium and water regimes**. New Phytol.

[39] Gibbs D.J., Coates J.C. (2014). AtMYB93 is an endodermis-specific transcriptional regulator of lateral root development in arabidopsis. Plant Signal Behav.

[40] Wang S., Alseekh S., Fernie A.R., Luo J. (2019). The structure and function of major plant metabolite modifications. Mol Plant.

[41•] Kiryushkin A.S., Ilina E.L., Guseva E.D., Pawlowski K., Demchenko K.N. (2022). Hairy CRISPR: genome editing in plants using hairy root transformation. Plants.

[42••] Voß L., Heinemann K.J., Herde M., Medina-Escobar N., Witte C.P. (2022). Enzymes and cellular interplay required for flux of fixed nitrogen to ureides in bean nodules. Nat Commun.

[43] Nakayasu M., Akiyama R., Lee H.J., Osakabe K., Osakabe Y., Watanabe B., Sugimoto Y., Umemoto N., Saito K., Muranaka T. (2018). Generation of α-solanine-free hairy roots of potato by CRISPR/Cas9 mediated genome editing of the St16DOX gene. Plant Physiol Biochem PPB.

[44] Aasim M., Katirci R., Baloch F.S., Mustafa Z., Bakhsh A., Nadeem M.A., Ali S.A., Hatipoğlu R., Çiftçi V., Habyarimana E. (2022). Innovation in the breeding of common bean through a combined approach of in vitro regeneration and machine learning algorithms. Front Genet.

[45] Niazian M., Belzile F., Torkamaneh D. (2022). CRISPR/Cas9 in planta hairy root transformation: a powerful platform for functional analysis of root traits in soybean. Plants.

[46] Shu H., Luo Z., Peng Z., Wang J. (2020). The application of CRISPR/Cas9 in hairy roots to explore the functions of AhNFR1 and AhNFR5 genes during peanut nodulation. BMC Plant Biol.

[47] Adachi K., Hirose A., Kanazashi Y., Hibara M., Hirata T., Mikami M., Endo M., Hirose S., Maruyama N., Ishimoto M. (2021). Site-directed mutagenesis by biolistic transformation efficiently generates inheritable mutations in a targeted locus in soybean somatic embryos and transgene-free descendants in the T1 generation. Transgenic Res.

[48] Zhang Z., Gao L., Ke M., Gao Z., Tu T., Huang L., Chen J., Guan Y., Huang X., Chen X. (2022). GmPIN1-mediated auxin asymmetry regulates leaf petiole angle and plant architecture in soybean. J Integr Plant Biol.

[49] Li X., Zhou H., Cheng L., Ma N., Cui B., Wang W., Zhong Y., Liao H. (2022). Shoot-to-root translocated GmNN1/FT2a triggers nodulation and regulates soybean nitrogen nutrition. PLoS Biol.

[50] Duan K., Cheng Y., Ji J., Wang C., Wei Y., Wang Y. (2021). Large chromosomal segment deletions by CRISPR/LbCpf1-mediated multiplex gene editing in soybean. J Integr Plant Biol.

[51] Carrijo J., Illa-Berenguer E., LaFayette P., Torres N., Aragão F.J.L., Parrott W., Vianna G.R. (2021). Two efficient CRISPR/Cas9 systems for gene editing in soybean. Transgenic Res.

[52] Yuan M., Zhu J., Gong L., He L., Lee C., Han S., Chen C., He G. (2019). Mutagenesis of FAD2 genes in peanut with CRISPR/Cas9 based gene editing. BMC Biotechnol.

[53] Neelakandan A.K., Wright D.A., Traore S.M., Ma X., Subedi B., Veeramasu S., Spalding M.H., He G. (2022). Application of CRISPR/Cas9 system for efficient gene editing in peanut. Plants.

[54] Wang L., Wang L., Tan Q., Fan Q., Zhu H., Hong Z., Zhang Z., Duanmu D. (2016). **Efficient inactivation of symbiotic nitrogen fixation related genes in*****Lotus japonicus*****using CRISPR-Cas9**. Front Plant Sci.

[55] Wang L., Rubio M.C., Xin X., Zhang B., Fan Q., Wang Q., Ning G., Becana M., Duanmu D. (2019). **CRISPR/Cas9 knockout of leghemoglobin genes in*****Lotus japonicus*****uncovers their synergistic roles in symbiotic nitrogen fixation**. New Phytol.

[56] Ji J., Zhang C., Sun Z., Wang L., Duanmu D., Fan Q. (2019). Genome editing in cowpea *Vigna unguiculata* using CRISPR-Cas9. Int J Mol Sci.

[57] Juranić M., Nagahatenna D.S.K., Salinas-Gamboa R., Hand M.L., Sánchez-León N., Leong W.H., How T., Bazanova N., Spriggs A., Vielle-Calzada J.P. (2020). **A detached leaf assay for testing transient gene expression and gene editing in cowpea (*****Vigna unguiculata*****[L.] Walp.)**. Plant Methods.

[58] Badhan S., Ball A.S., Mantri N. (2021). First report of CRISPR/Cas9 mediated DNA-free editing of 4CL and RVE7 genes in chickpea protoplasts. Int J Mol Sci.

[59] Miller S.S., Dornbusch M.R., Farmer A.D., Huertas R., Gutierrez-Gonzalez J.J., Young N.D., Samac D.A., Curtin S.J. (2022). **Alfalfa (*****Medicago sativa*****L.)*****pho2*****mutant plants hyperaccumulate phosphate**. G3 Genes, Genomes, Genet.

